# Pharmacogenomic Characterization in Bipolar Spectrum Disorders

**DOI:** 10.3390/pharmaceutics12010013

**Published:** 2019-12-21

**Authors:** Stefano Fortinguerra, Vincenzo Sorrenti, Pietro Giusti, Morena Zusso, Alessandro Buriani

**Affiliations:** 1 Maria Paola Belloni Center for Personalized Medicine, Data Medica Group (Synlab Limited), 35131 Padova, Italy; stefano.fortinguerra@gmail.com (S.F.); vincenzosorrenti88@gmail.com (V.S.); 2 Department of Pharmaceutical & Pharmacological Sciences, University of Padova, 35131 Padova, Italy; pietro.giusti@unipd.it (P.G.); morena.zusso@unipd.it (M.Z.); 3 Bendessere™ Study Center, Solgar Italia Multinutrient S.p.A., 35131 Padova, Italy

**Keywords:** personalized medicine, bipolar disorder, antipsychotics, mood stabilizers, pharmacogenomics

## Abstract

The holistic approach of personalized medicine, merging clinical and molecular characteristics to tailor the diagnostic and therapeutic path to each individual, is steadily spreading in clinical practice. Psychiatric disorders represent one of the most difficult diagnostic challenges, given their frequent mixed nature and intrinsic variability, as in bipolar disorders and depression. Patients misdiagnosed as depressed are often initially prescribed serotonergic antidepressants, a treatment that can exacerbate a previously unrecognized bipolar condition. Thanks to the use of the patient’s genomic profile, it is possible to recognize such risk and at the same time characterize specific genetic assets specifically associated with bipolar spectrum disorder, as well as with the individual response to the various therapeutic options. This provides the basis for molecular diagnosis and the definition of pharmacogenomic profiles, thus guiding therapeutic choices and allowing a safer and more effective use of psychotropic drugs. Here, we report the pharmacogenomics state of the art in bipolar disorders and suggest an algorithm for therapeutic regimen choice.

## 1. Introduction

One important and recent development in the area of mood disorders is the recognition that many patients initially suspected suffering from major depressive disorder suffer instead from a form of bipolar disorder (BD) [1]. Symptomatic bipolar disorder patients are more frequently in the depressive condition rather than in hypomanic, manic, or mixed states [1,2,3]. As a consequence, such patients can be improperly diagnosed as suffering from major depression and prescribed antidepressants instead of the lithium, atypical antipsychotics, or antiepileptic-type mood stabilizers normally used to treat bipolar spectrum disorders [2,3]. Up to 50% of the patients once identified as suffering from unipolar depression are now recognized to be suffering from a bipolar spectrum disorder [1,2,3]. Antidepressant treatment of unrecognized bipolar patients can increase the cyclical nature of the mood disorder, mixed states, and conversion to hypomania and mania, and can also contribute to increase suicide in younger patients (<25 years) [4,5,6]. The World Mental Health Survey Initiative estimated a 12 month and total lifetime prevalence of 1.5% and 2.4%, respectively, for bipolar disorder I, bipolar disorder II, and subthreshold BD [5,7]. Most people are in their teens or early twenties when bipolar disorder symptoms begin. The diagnosis of one of the various bipolar spectrum disorders is very complex, especially during the early disease stages. It is estimated that only 20% of patients with one of the bipolar spectrum disorders associated with depressive episodes are diagnosed and treated correctly within one year, with a 5–10 years delay typical between the onset of symptoms and a BD diagnosis [8]. The main problem in making a diagnosis is distinguishing between bipolar I and II disorders from unipolar depression, especially when patients do not have a clear history of episodes of hypomania or mania.

**Figure 1:**
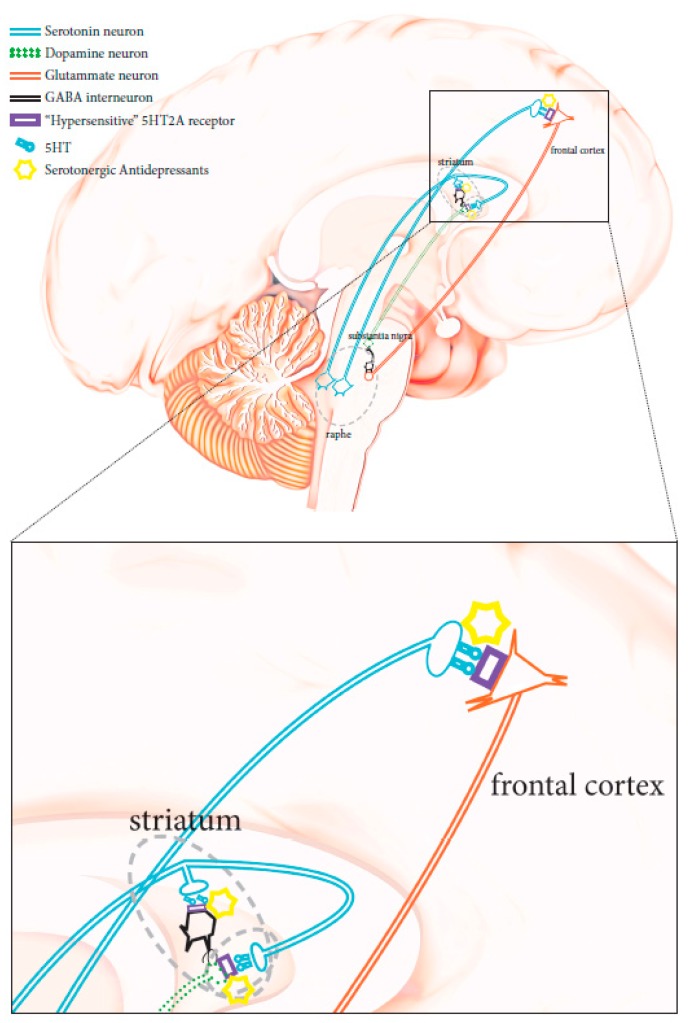
Schematic diagram showing neuronal CNS pathways involved in mania-inducing side effects caused by the administration of a serotoninergic antidepressant when *HTR2A* is mutated (see text for more details).

The physiopathology of manic–depressive illness (MDI), or bipolar disorders (BPD) has not been completely clarified. No objective biological marker is available to determine with precision the state of the disease. However, twins, family, and adoption studies have highlighted the association of genetic components with the condition. First-degree relatives of subjects suffering from bipolar disorders are about seven times more likely to develop the disease compared to the general population, while the heritability of bipolar I disorder has been recently estimated at 0.73 [9]. Unipolar depression has been reported as the main misdiagnosis in patients affected by bipolar II disorder, as these patients do not have episodes of complete mania and have mainly depressive episodes, as opposed to hypomania [5,7]. According to the current diagnostic criteria, the distinction of a depressed patient between unipolar major depressive disorder and bipolar spectrum disorder is not simple and is solely entrusted to the clinic. As for other psychiatric conditions, biological parameters are lacking, although some molecular processes conceivably associated with bipolar conditions have been suggested, particularly CNS serotonergic pathway alterations [10,11]. *HTR2A* gene variations have been shown to affect serotonergic receptors, making them hypersensitive to serotonin. When triggered, these hypersensitive receptors could abnormally stimulate glutamatergic transmission Figure 1 [12,13,14], thus contributing to the induction of states of mania, agitation, and anxiety. Therefore, the use of serotonergic antidepressant therapies can lead to the degeneration of a bipolar disorder spectrum condition [2,3,7]. Unrecognized bipolar patients carrying such genetic mutations have a higher risk of developing a full-blown bipolar condition when receiving serotonergic antidepressants, and should avoid the use of these drugs [15].

## 2. Clinical Classification of Bipolar Spectrum Disorders

In general, bipolar disorders are mood disorders that, unlike depressive disorders, which are characterized by a single polarity, present manic or hypomanic episodes alternating with depressive episodes. They are generally divided into:

- Bipolar I disorder (BDI): The characteristic that primarily characterizes this condition is alternating manic and depressive episodes. BDI is characterized by the appearance of one or more manic or mixed episodes (an overt phase of mania concomitant with a full-blown phase of depression) lasting at least a week. Patients with BDI can also experience episodes of major depression.
- Bipolar II disorder (BDII): This condition is characterized by alternating depressive and hypomanic episodes. Type II bipolar disorder is a mental disease similar to type I bipolar disorder, with moods that cycle between highs and lows, although in BDII the “highs” never reach a complete mania state (hypomania). Subjects with bipolar II disorder suffer more frequently from depressive episodes than from hypomania. Given that hypomania can be confused with normal happiness or even normal functioning, bipolar II disorder can often be misdiagnosed as a unipolar depression.
- Cyclothymia: This condition is characterized by the alternation of mild depressive episodes and mild hypomanic episodes. In cyclothymia, a person has hypomania (as in bipolar II disorder) that frequently alternates with short periods of mild depression. When present, however, the symptoms of depression do not last long.
- Bipolar disorder not otherwise specified (more recently labeled “not elsewhere classified”): in bipolar disorder not elsewhere classified, people have symptoms of mania or hypomania that are too low or too brief to meet the diagnostic criteria for a syndrome.

Currently, the diagnostic characterization of bipolar spectrum disorders is based mostly on clinical observations, family history, and collection of personal information from those closest to the patient [2]. Symptoms suggesting that depression may be part of a bipolar disorder include increased sleep duration, psychomotor retardation, hyperphagia, psychotic symptoms, anxiety, suicidal ideation, and emotional lability during episodes [2,3]. Other signs that may orient diagnosis towards a bipolar depression are related to the course of the disease, including a high frequency of depressive symptoms, an early age of onset, a high number of sick days, and a sudden symptomatic reduction or disappearance [2,5]. The response to antidepressant treatment can also suggest a bipolar depression, such as a sequence of failures with antidepressants and the appearance of side effects like anxiety, agitation, and insomnia [2,3,5].

In the DSM-5 [16], bipolar disorders and related conditions have been distinguished from depressive disorders and relocated between psychotic and depressive disorders, as an intermediate group of conditions in terms of genetics, family history, and symptomatology. Conditions classified as bipolar disorders comprise a range of diagnostic subgroups that can be characterized by the severity of the mood alterations experienced in the acute phase [5].

## 3. Pharmacological Treatments of Bipolar Spectrum Disorders

Before the advent of lithium, common treatments for bipolar patients were solanaceae alkaloids, bromides, and, starting from the early 1900s, barbiturates. The efficacy of lithium salts in the treatment of mania was postulated in 1800s [17]. The antimanic effect was later demonstrated in the 1950s, and it was only in the late 1960s that its effectiveness was demonstrated in bipolar disorder for the prophylaxis of manic–depressive episodes [18]. However, the FDA (US Food and Drug Administration) only released the authorization for its antimanic use in 1978 [19], limited to maintenance/prophylaxis of manic–depressive episodes. In 1995, the FDA approved the anticonvulsant valproic acid for its antimanic use. At the same time, the antiepileptic carbamazepine was introduced for the treatment of bipolar disorder, with approval from both the FDA and NICE (National Institute for Health and Care Excellence). Since 2000, the FDA has approved several atypical antipsychotic drugs (AADs) for their antimanic action (quetiapine, aripiprazole, olanzapine, risperidone, ziprasidone, etc.), or for their antidepressant action (lurasidone, quetiapine). Finally, lamotrigine, a more recent antiepileptic drug, has been approved in bipolar disorder for the prevention of depressive episodes [20,21,22].

### 3.1. Lithium

Lithium is used to decrease the recurrence of manic episodes, but it is also indicated for depressive episodes, although to a lesser extent [5]. Even though lithium has been used in the treatment of bipolar disorders for almost 60 years, its mechanism of action has not been fully clarified [23]. Some molecular steps have been implicated along the signal transduction cascade activated by neurotransmitter receptors, such as G proteins and phosphatidyl inositol. More recently, gene expression regulation of growth factors and neuronal plasticity has been associated with lithium activity via components of signal transduction, including protein kinase C and GSK3 [5,23,24]. Based on these and other putative molecular associations of the lithium pathways, several studies have been carried out in order to identify potential genetic lithium response predictors [25].

### 3.2. Antiepileptics as Mood Stabilizers

Based on the theory that the recurrence of manic episodes can expose the subject to further manic episodes (kindling), a logical parallelism has been drawn with epilepsy, where the appearance of repeated epileptic seizures exposes the subject to further seizures [5,26,27]. Several antiepileptics are often prescribed to treat bipolar disorders, some more effectively than others [26,27].

#### 3.2.1. Valproic Acid

Valproate is recommended in long-term treatment to prevent mania recurrence in bipolar disorder. It is also used in the acute phase of mania, although its preventive activity has not been adequately clarified in this context [28]. Like lithium, valproic acid can be administered once a day with other mood stabilizers at doses that correspond to the lower limit of the therapeutic range, to enhance tolerability and therapeutic compliance [29]. As with all antiepileptics, its precise mechanism of action is uncertain. At least three hypotheses have been proposed: inhibition of the voltage-dependent sodium channel, enhancement of GABAergic neurotransmission, and regulation of downstream signal transduction cascades [28,29]. To explain its mood-stabilizing activity in mania, it has been hypothesized that valproate acts by reducing the excessive stimulation of neurotransmission, inhibiting the ionic flow through the voltage-dependent sodium channels (VSSC) [28]. No specific molecular site has been identified, but the drug may alter the phosphorylation of sodium channels, thus modifying their sensitivity. When less sodium enters the neuron, there is a reduction in glutamate release and therefore excitatory neurotransmission [28,29,30]. Others have hypothesized that valproate could enhance GABA activity by decreasing its re-uptake, increasing its release, or slowing its metabolic degradation. Although it remains unknown exactly how the enhancement of GABAergic tone is achieved, it is believed that this may explain the antimanic effect of valproic acid [28]. More recently, further mechanisms have been proposed for valproate that could explain its activity. Valproate can inhibit GSK3, but it can act on other molecular targets too; it can inhibit MARCKS (substrate of miranolated kinase C rich in alanine) as well as protein kinase C (PKC), and can activate various signals that promote long-term neuroprotection, such as BCL2, GAP43, ERK, and others [28,29,30].

#### 3.2.2. Carbamazepine

Carbamazepine was the first antiepileptic with demonstrated effectiveness for mania in bipolar disorder [5,31,32], although it did not formally receive FDA approval. It is hypothesized that carbamazepine acts by blocking the voltage-dependent sodium channels (VSSC), possibly at the VSSC subunit level within the channel [33].

#### 3.2.3. Lamotrigine

Lamotrigine acts as a mood stabilizer and it is used in prevention of depression and mania, although this use has not been approved by FDA in bipolar depression. Nonetheless, in many guidelines on treatment of bipolar depression, this drug is preferred to antidepressants as a first-line drug [5,34]. The reduction of excitatory-type glutamatergic neurotransmission may represent the specific mechanism of action of lamotrigine [35]. Some other antiepileptic drugs, like gabapentin, topiramate, oxcarbazepine/eslicarbazepine, and pregabalin, some calcium channel blockers of type L (e.g., dihydropyridine), and riluzole, are sometimes prescribed in “experimental” treatments for symptoms associated with bipolar disorder [5].

### 3.3. Atypical Antipsychotics

Atypical antipsychotics have been shown to be effective for the main non-psychotic symptoms of mania and for the prevention of recurrence of mania. Currently, they are the most effective therapeutic option for bipolar disorder, along with most antiepileptics and lithium [5,36]. The mechanism of action of atypical antipsychotics in bipolar disorder is not yet fully understood, but the prevailing hypothesis is that antagonism or partial agonism of D2 receptors could explain the reduction of manic psychotic symptoms. Moreover, the 5HT2A receptor (5HT2AR) antagonism and partial agonism of 5HT1A receptors could be responsible for the reduction of manic and non-psychotic depressive symptoms observed with some atypical antipsychotics. This may be achieved through the downregulation of the glutamatergic system affecting pyramidal neurons. Given that hyperactivity of the glutamatergic system, depending on the neuronal circuit involved, can be associated with both manic and depressive symptoms, these antipsychotics can be effective in reducing both types of symptoms. Other mechanisms have also been hypothesized to explain why some atypical antipsychotics improve the symptomatic picture of the depressive phase of bipolar disorder. All these mechanisms are based on the ability of some atypical antipsychotics to increase serotonin, dopamine, and norepinephrine levels, while reducing those of glutamate [37,38]. Atypical antipsychotics are indicated in schizophrenia, and most of them also in mania, but quetiapine is the only one approved for bipolar conditions, while lurasidone has only been tested [39,40].

### 3.4. Benzodiazepines

Although the use of these drugs as mood stabilizers has not been formally approved, benzodiazepines represent a treatment of considerable value, especially during emergencies. Their prompt administration can provide an immediate sedative effect and provide precious time, unlike mood stabilizers with a slower onset of action. Benzodiazepines are essential drugs for patients who suffer from intermittent episodes of agitation, insomnia, and incipient manic symptoms and require treatment as needed [41].

### 3.5. Antidepressants

Evidence is growing that antidepressants in these disorders not only do not work, but can even exacerbate the condition of some patients with bipolar disorder, leading to mania and hypomania states, destabilizing mood, and increasing cyclicality or even suicidality [2,3,42,43].

Evidence suggests that dysfunctions of glutamate neurotransmission may be implicated in various psychiatric conditions, including bipolar disorders [44,45]. The role of 5HT-mediated glutamatergic activation in BD could explain why the use of antidepressants can exacerbate manic symptoms in bipolar disorders. This hypothesis is still under study, but little doubt remains that antidepressants, tricyclic ones in particular, can trigger manic symptoms in subjects with bipolar spectrum disorders. 5HT2ARs are always postsynaptic and are located in many brain regions. In cortical neurons, they are coupled with Gαq/11 type G proteins. The latter activates membrane-bound phospholipase C beta, leading to cleavage of PIP2 into two messengers, IP3 and diacylglycerol (DAG). This stimulates protein kinase C (PKC), which in turn controls the function of the main glutamate transporter in CNS, Glutamate transporter-1 [46,47,48]. In particular, PKC-mediated phosphorylation induces GLT1 transporter downregulation/endocytosis, thus increasing glutamatergic intersynaptic activity [49,50,51,52].

### 3.6. Associations

In clinical practice, many subjects suffering from bipolar disorder need to be treated with more than one drug. Effective combinations include the association of valproate or lithium with an atypical antipsychotic. Evidence collected from clinical practice suggests more associations can be utilized, although such suggestions have not been adequately evaluated in controlled clinical trials. Examples include the combination of valproate and lithium, valproate and lamotrigine, lithium and lamotrigine, lithium with quetiapine, and lamotrigine with valproate and lithium. Expert opinions are very divergent when it comes to treating bipolar depression, particularly with antidepressants. Some believe that an antidepressant should not be given in any case, while others simply recommend caution when combining an antidepressant with a mood stabilizer.

In conclusion, current protocols and guidelines for acute mania recommend as first-line treatments lithium, quetiapine, divalproex, asenapine, aripiprazole, paliperidone, risperidone, and cariprazine in combination or alone. In bipolar I depression, recommended first-line treatments include quetiapine, lurasidone plus lithium or divalproex, lithium, lamotrigine, lurasidone, or adjunctive lamotrigine. Except for antidepressants, treatment with drugs that have shown efficacy in the acute phase is also recommended in the maintenance phase. For patients initiating or switching drugs in the maintenance phase, the use of monotherapy or combinations of lithium, quetiapine, divalproex, lamotrigine, asenapine, and aripiprazole can be suggested [5,53].

## 4. Genetics of Bipolar Spectrum Disorders

Multiple genetic studies have pointed out that bipolar disorders (BPD) are often heritable conditions, with genetics accounting for 60–85% of the risk [54,55]. Studies indicate that the risk of recurrence of bipolar disorders in first-degree relatives is about 9%, almost 10-fold higher than in the general population [55,56]. Family studies have also indicated that bipolar I and II disorders have a genetic distinction; the risk of bipolar II disorder among relatives of patients with bipolar II disorder is greater than in relatives of patients with BDI [57,58,59].

Research on genes that might influence bipolar disorders has been hampered by the phenotypic and genetic complexity of the syndrome, a limited knowledge on its pathogenesis, and by the scarcity of animal models. Linkage studies have highlighted different chromosomal regions as carriers of meaningful genes, but with inconsistent results. It is now generally recognized that the genetic associations of bipolar disorder are linked to many different genes [60,61]. Thus, genetic research in the field has been focused on genome-wide association studies. The application of this approach to BD since 2007 has allowed the identification of a sizable number of candidate genes, which have since been associated with the disorder in various studies (including *DAOA*, *BDNF*, *GRIK4*, *DISC1*, *TPH2*, and *SLC6A4)* [62]. More recently, the first major BD genome-wide association study by the Psychiatric Genomics Consortium (PGC) Bipolar Disorder Working Group led to the identification of four significant loci at the genomic level. The study analyzed 7481 BD patients and compared them to 9250 controls. Three subsequent meta-analyses that included PGC BD data identified five more loci [63]. In one of the most important studies on the subject, a genome-wide association study was conducted with 20,352 cases compared to 31,358 controls. A total of 822 sentinel variants were followed up independently in 9412 cases versus 137,760 controls. As a result, 30 loci (with 20 new ones) achieved significant genomic association evidence and contained genes coding for synaptic components (*ANK3*, *RIMS1*) and transporters, neurotransmitters and ion channels (*SLC4A1*, *CACNA1C*, *SCN2A*, *GRIN2A*). Interestingly type I BD is genetically highly correlated with schizophrenia, while type II bipolar disorder correlates more with major depressive disorder [62,63]. In summary, the results of the broader genomic analysis on BD have revealed that these conditions have an extensive polygenic architecture, implicating in their etiology neurotransmitter and calcium channel functions, confirming that BD falls within a spectrum of highly related psychiatric disorders [55]. The detailed genetic dissection of the disorder, rationalized using a systems biology approach, could allow the identification of functional connections between the different molecular effectors identified, leading to the construction of a molecular network underlying the pathogenesis of the disease. This could also represent a valuable tool to guide, optimize, and personalize the therapeutic choice of molecular targets.

## 5. Pharmacogenomics of Bipolar Spectrum Disorder

The study of pharmacogenomics in the field of mental health is rapidly growing. Most data on genomics of bipolar spectrum disorders concentrate on the variability (response, side effects) of the pharmacological response when using atypical antipsychotics and/or antidepressants [36,64]. The genetic assets used in clinical settings are based mostly on the pharmacogenomic studies associating gene polymorphisms with treatment outcomes. In particular, genetic polymorphisms can affect pharmacodynamic and pharmacokinetic aspects of medications, thus influencing efficacy and susceptibility to side effects.

With the increased accessibility of individual DNA chip analysis, WES (whole-exome sequencing,) and WGS (whole-genome sequencing) for diagnostic studies [65], pharmacogenomics is entering a phase of regular clinical use. The analysis of some key point mutations is actually required before specific drugs can be administered, in order to personalize the treatment according to the predicted efficacy or sensitivity to side effects [66]. Genetic variants to be analyzed for each class of drugs are selected according to available evidence-based medicine data and clinical validations, and several dedicated databases are available and freely accessible on the web today (e.g., pharmGKB [67], drugbank [68], genecards [69]).

Several factors can increase the possibility of a genetic diathesis in the treatment of bipolar spectrum disorders. Some meaningful SNPs are used in clinical settings to predict therapeutic response or potential toxicity. Polygenic determinants of drug effects have become more and more important in pharmacogenomics and are now used in clinical diagnostics to prevent adverse reactions to medications and to optimize therapy. Certain aspects of pharmacogenomic testing have entered the clinical routine. For example, FDA recommends testing for HLA-B*1502 when using carbamazepine. Carriers of this mutation are estimated to have a 10-fold higher risk for Stevens–Johnson syndrome when assuming the drug [70]. Today, the majority of tests available include both pharmacodynamic (PD) and pharmacokinetic (PK) genomic analytical panels.

### 5.1. Pharmacogenomics of Pharmacodynamic Pathways

Examples of genes implicated in the pharmacodynamics of neuropsychiatric drugs include *COMT*, *DRD2* dopamine receptor, *HTR2C* and *HTR2A* serotonin receptors, and *SLC6A4* serotonin transporter [71,72,73,74].

Serotonergic pathways have been the focus of most pharmacogenetic studies on clinical response to psychotropic drugs in BD patients. Alterations of these pathways have been implicated in bipolar disorders. The serotonin transporter *SLC6A4* gene has been extensively investigated. *5-HTTLPR* is a functional polymorphism of *SLC6A4*, with a short (s) and a long (l) allele variant that have been suggested to be related to stress and psychiatric disorders. There have been reports associating the (s) allele with poor response to serotonergic drugs in BD. On the other hand, genetic studies on the (l) allele and SSRI response have not been able to clearly demonstrate a functional association between gene expression and pharmacological effect. For example, rs25531, a SNP located in the same *SLC6A4* region as *5-HTTLPR*, although associated with increased transporter expression, does not affect the response to SSRIs [75,76].

The *HTR2A* gene for 5HT-2A receptors (5HT2ARs) has been associated with antidepressant effect. Patients with specific polymorphisms of this gene (rs6313 and rs7997012) have been found to respond better to antidepressants. The same mutations have also been associated with an increased sensitivity of 5HT2ARs to serotonin [77,78]. 5HT2ARs hypersensitivity found in rs6313 and rs7997012 genetic mutations has been associated with the occurrence of pharmacologically induced dysphoric conditions in misdiagnosed bipolar spectrum disorders [15]. Several studies have shown an association between a good response to drugs that act on the serotonergic pathway in BD patients, and the rs6295 C/C genotype in the *HTR1A* gene [79,80,81]. The *HTR2C* gene for the 5HT-2C receptor has been shown to be associated with adverse drug reactions when using neuroleptic drugs. There is evidence indicating an association of specific polymorphisms like variant rs3813929 with a higher risk of extrapyramidal side effects. Another polymorphism, rs1414334, has been associated with higher risk of developing metabolic syndrome in subjects treated with olanzapine [82,83].

Dopamine 2 receptors have been associated with antipsychotic effect and are coded by the highly polymorphic *DRD2* gene, located on chromosome 11q22. An extensively studied variant is -141C Ins/Del (rs1799732). When the Ins/Ins genotype is present, patients respond better to antipsychotic drugs than subjects carrying one or two copies of the Del allele. Subjects carrying the homozygous C allele with rs2514218 have been shown to respond better to antipsychotics than those homozygous for T allele, but they also present more side effects [84,85,86].

Little evidence is available on genes encoding for glutamate receptors. Research has been focused on the *Gβ3* gene, with studies reporting an association between a good response to drugs that act on the serotoninergic pathway in BD patients, and the T/T genotype in the rs5443 polymorphism [79,87].

Table 1 shows some of the most important pharmacodynamic mutations that can modify the pharmacotoxicological outcomes of drugs in bipolar disorder.

### 5.2. Pharmacogenomics of Pharmacokinetic Pathways

CYP enzyme polymorphisms (cytochrome P450) may result in increased or decreased enzymatic activity. Such variations can thus determine a wide variety of drug metabolic patterns that, depending on the resulting metabolic activity, characterize the subject as a normal metabolizer, ultra-rapid metabolizer, intermediate metabolizer, or poor metabolizer. Poor metabolizers are subjects that have poor to no enzymatic activity, usually associated with having two copies of non-functioning alleles. Medications targeted by these enzymes are not metabolized effectively, thus increasing the risk of adverse drug reactions. In the case of pro-drugs, this condition can result in therapeutic failure, since the medication will not be transformed into the active form. Intermediate metabolizers have a slight functional impairment of drug metabolic activity due to at least one non-functioning allele. Normal metabolizers have a regular drug metabolic activity, while ultra-rapid metabolizers have enhanced enzyme activity that leads to an increased ability to metabolize drugs, decreasing the effectiveness of drugs, or increasing the risk of adverse drug reactions of prodrugs. For reference, the Clinical Pharmacogenetics Implementation Consortium (CPIC) published a review on pharmacogenomics nomenclature [161,162].

The drug metabolism (phase I) of psychiatric medications involves CYP 1 to three families, including CYP1A2, CYP2B6, CYP3A4/5, CYP2C9, CYP2C19, and CYP2D6. These CYP enzymes can be analyzed to personalize drug prescription and administration [163].

The FDA labeling (US Food and Drug Administration) of about 200 drugs specify their pharmacogenomic characterization. Neuropsychiatric medications represent one fourth of all these drugs. With pharmacogenomic information on the type of metabolizer, it is possible to choose the most effective drug for a patient, and identify the dose range and the administration strategy.

Table 2 shows some of the most important pharmacokinetic mutations that can modify the pharmacotoxicological outcome of the drugs in bipolar disorder.

## 6. Conclusions

Bipolar spectrum disorder diagnosis is usually not based on the identification of pathogenetic mechanism, but on symptoms and signs, while the identification and association of traditional biological markers with bipolar disorder is still under investigation. The advent of genomics has allowed the identification of genetic assets associated with bipolar spectrum disorders, providing the basis for the identification of genetic risk factors and the definition of personalized pharmacotoxicological profiles. These can guide the initial therapeutic choice, or suggest corrections according to the individual’s biological networks implicated in the disease pathways or in the relevant pharmacological aspects.

Even before they are diagnosed with a bipolar condition, patients usually claim a depressive symptomatology of some degree. Using the patient’s molecular information can be helpful for the initial therapeutic orientation when a decision needs to be made on whether to use serotonergic antidepressants, given that mutations in their *HTR2A* gene would suggest a risk of manic state induction.

Once the bipolar condition is diagnosed, pharmacogenomic information can be used to guide the choice of the class of drugs to be used. In particular, the analysis of pharmacodynamically relevant SNPs can support the identification of the type of drugs with most chance of being effective and/or least likelihood of causing side effects. This information should be combined with a complete family history and medical information, as well as regular clinical risk factor profiling for bipolar disorder.

When the type of drug class to be used has been identified, pharmacogenomic information can help to drive the choice of the best molecule according to the individual pharmacokinetic asset, and at the same time indicate the dose and the therapeutic strategy that should be used to optimize effectiveness and minimize the risks of side effects.

**Figure 2:**
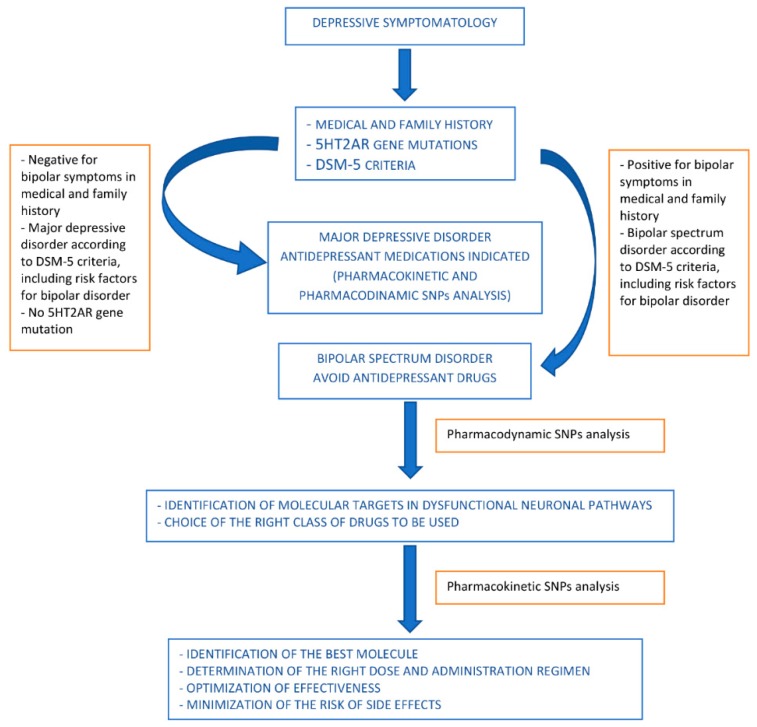
Proposed algorithm for drug use orientation in pharmacological treatment of bipolar spectrum disorders.

A proposed algorithm is shown in Figure 2.

In summary, the genomic knowledge available today can support a personalized medicine approach to bipolar disorders, suggesting the most suitable pharmacological therapy for each patient. Together with the basic clinical information, a pharmacogenomic analysis should always be recommended to verify how one or more drugs might effectively act in a given subject, foreseeing the efficacy profile and safety of each drug, in order to increase therapeutic success and decrease unwanted adverse effects. Although more evidence is required before genomic data can be fully used to assist the clinician with a molecular diagnosis, when dealing with bipolar spectrum disorders, pharmacogenomics can provide orientation towards a safer use of drugs, particularly antidepressant drugs, based on the polymorphisms on the *HTR2A* gene. Once a bipolar disorder is diagnosed, drugs can be chosen based on the molecular targets identified by the pharmacodynamic SNPs, optimizing the doses and the pharmacological combinations based on the pharmacokinetic SNPs.

## Figures and Tables

**Table 1:** SNPs relevant for the pharmacodynamics of drugs used for treatment of bipolar spectrum disorders.

Gene	Protein	SNPs	Drugs	Results	Level of Evidence (1A Strongest)	Ref.
*ADCY1*	Adenylate cyclase 1	rs1521470	lithium	Patients with the AA genotype and bipolar affective disorder may have a decreased response to lithium as compared to patients with the AG or GG genotypes.	3	[88]
*ADCY2*	adenylate cyclase 2	rs1544938 rs4702484	antipsychotics	Patients with the CC genotype may have increased response to antipsychotics compared to patients with the GG genotype.	3	[89]
*ADRB2*	Adrenoceptor beta 2	rs1042713 rs8050896	risperidone paliperidone	Patients with the GG or AG genotype may have increased likelihood of sexual adverse events when treated with risperidone as compared to patients with the AA genotype.	3	[90,91]
				Patients with the TT genotype may have an increased response to risperidone as compared to patients with the AA or AT genotypes.	3	
*AKT1*	AKT serine/threonine kinase 1	rs2494732	risperidone paliperidone	Patients with the TT may have an increased response to risperidone as compared to patients with the CC or CT genotype.	3	[92]
*ANKK1*	Ankyrin repeat and kinase domain containing 1	rs1800497	valproic acid aripiprazole risperidone paliperidone	Patients with the AA genotype may have increased risk of side effects including hyperprolactinemia and weight gain, but decreased risk of tardive dyskinesia, as compared to patients with the AG or (GG: increased risk of tardive dyskinesia) genotype.	2B	[93]
*ASIC2*	Acid sensing ion channel subunit 2	rs11869731	lithium	Patients with the CC genotype may have a better response to lithium.	3	[94]
*BDNF*	Brain derived neurotrophic factor	rs6265 rs11030104	antipsychotics antidepressants	Patients with the AA genotype and schizophrenia may show less resistance to treatment with antipsychotics as compared to patients with the AG or GG genotype.	3	[25,75,95]
				Patients with the CC genotype and depressive disorder may be more likely to respond to paroxetine but less likely to respond to citalopram or antidepressants as compared to patients with the CT or TT genotype.	3	
*CACNG2*	Calcium voltage-gated channel auxiliary subunit gamma 2	rs2284018 rs2284017	lithium	Patients with the CC or CT genotype may be more likely to respond to lithium.	3	[96,97]
*CCL2*	C-C motif chemokine ligand 2	rs4586	risperidone paliperidone	Patients with the GG genotype and schizophrenia may have a poorer response when treated with risperidone as compared to patients with the AA or AG genotype.	3	[98]
*CNR1*	Cannabinoid receptor 1	rs1049353 rs806378	aripiprazole clozapine haloperidol olanzapine quetiapine risperidone	Patients with the CC genotype and psychotic disorders may have an increased likelihood of weight gain as compared to patients with the CT and CC genotypes.	3	[99]
*COMT*	catechol-O-methyltransferase	rs4818 rs4680 rs13306278	antipsychotics SSRI	Patients with the GG genotype may have a better response to treatment.	3	[100,101]
				Patients with the AA genotype may have increased blood pressure when treated with antipsychotics as compared to patients with the GG genotype.	3	
				Patients with the CC genotype may have increased likelihood of remission when treated with Selective serotonin reuptake inhibitors compared to patients with the TT or CT genotype.	2B	
*CPS1*	Carbamoyl-phosphate synthase 1	complete gene sequencing	valproic acid Testing suggested by FDA and PMDA	Valproic acid is contraindicated in patients with known urea cycle disorders (UCDs), due to a risk for severe hyperammonemia. UCDs result from mutations in one of several genes, such as carbamoyl-phosphate synthetase 1 (CPS1) deficiency.	none	[102]
*DRD1*	Dopamine receptor D1	rs4532	lithium	Patients with the TT genotype may have an increased response to lithium as compared to patients with the CC genotype.	4	[103]
*DRD2*	Dopamine receptor D2	rs1800497 rs1799978	antipsychotics	Patients with the AA genotype may have increased risk of side effects including hyperprolactinemia and weight gain, but decreased risk of tardive dyskinesia, during treatment with antipsychotic drugs as compared to patients with the AG or GG genotype.	2B	[93,104,105]
*DRD3*	Dopamine receptor D3	rs6280	quetiapine	People with TT genotype may have increased clearance of quetiapine compared with people with genotypes CC or CT.	3	[106,107]
*EPM2A*	EPM2A, laforin glucan phosphatase	rs1415744	chlorpromazine clozapine haloperidol olanzapine quetiapine risperidone	Patients with the CC genotype and schizophrenia may have increased response to chlorpromazine, clozapine, haloperidol, olanzapine, quetiapine, and risperidone compared to patients with the CT and TT genotypes.	3	[108]
*FAAH*	Fatty acid amide hydrolase	rs324420	aripiprazole clozapine haloperidol olanzapine quetiapine risperidone	Patients with the AA genotype and psychotic disorders who are treated with aripiprazole, clozapine, haloperidol, olanzapine, quetiapine, or risperidone may have an increased likelihood of weight gain of more than 7% of baseline body weight as compared to patients with the CC genotype.	3	[109,110,111,112]
*FAM177A1*	Family with sequence similarity 177 member A1	rs79403677	lithium	Patients with the GG genotype and bipolar affective disorder may have an increased response to lithium as compared to patients with the GT or TT genotypes.	3	[88]
*FAM178B*	Family with sequence similarity 178 member B	rs6728642	lithium	Patients with the AA genotype and bipolar affective disorder may have a decreased response to lithium as compared to patients with the AG or GG genotypes.	3	[88]
*FKBP5*	FKBP prolyl isomerase 5	rs1360780	clomipramine lithium paroxetine venlafaxine	Patients with the CC genotype may (1) have decreased response to antidepressants (2) have decreased, but not absent, risk for suicide ideation with paroxetine, venlafaxine, clomipramine, and lithium, as compared to patients with the CT or TT genotype.	2B	[103]
*GABRA1*	Gamma-aminobutyric acid type A receptor alpha1 subunit	rs2279020	carbamazepine phenytoin valproic acid	Patients with the GG genotype treated with antipsychotics may have increased risk for drug-resistance as compared to patients with the AA genotype.	3	[113]
*GADL1*	glutamate decarboxylase like 1	rs17026688	lithium	Allele T is associated with increased response to lithium when treated with lithium in people with bipolar disorder as compared to allele C.	none	[90,114]
*GNB3*	G protein subunit beta 3	rs5443	risperidone paliperidone olanzapine	Patients with the CC genotype and schizophrenia who are treated with olanzapine may have a decreased, but not absent, risk of weight gain as compared to patients with the CT or TT genotype.	3	[115]
*GRAMD1B*	GRAM domain containing 1B	rs61123830	lithium	Patients with the AA genotype and bipolar affective disorder may have a decreased response to lithium as compared to patients with the AG or GG genotypes.	3	[88]
*GRID2*	Glutamate ionotropic receptor delta type subunit 2	rs1875705	risperidone paliperidone	Patients with the GG genotype may have an increased response to risperidone as compared to patients with the AA and AG genotypes.	3	[116]
*GRIN2B*	Glutamate ionotropic receptor NMDA type subunit 2B	rs1806201 rs1019385 rs1072388	risperidone quetiapine valproic acid clozapine	Patients with the GG genotype who are treated with risperidone may have an increased likelihood of adverse reactions as compared to patients with the AA or AG genotype.	3	[117]
				Patients with the AA genotype who are treated with quetiapine may have an increased likelihood of neurological adverse reactions and sleepiness as compared to patients with the AG or GG genotype.	3	
				Patients with the CC genotype and epilepsy may require a decreased dose of valproic acid as compared to patients with the AA or AC genotype.	3	
				Patients with the GG genotype and schizophrenia may have a worse response when treated with clozapine as compared to patients with the AA or AG genotype.	3	
*GRM3*	Glutamate metabotropic receptor 3	rs724226	risperidone paliperidone	Patients with the GG genotype who are treated with risperidone may have more improvement in symptoms as compared to patients with the AA genotype.	3	[118]
*GRM7*	Glutamate metabotropic receptor 7	rs2069062	risperidone paliperidone	Patients with the CC genotype may have increased response to risperidone as compared to patients with the CG and GG genotypes.	3	[116]
*GSK3B*	Glycogen synthase kinase 3 beta	rs334558 rs6438552	lithium	Patients with the AA genotype and bipolar disorder may be less likely to respond to lithium as compared to patients with the GG or AG genotype.	3	[119]
*HLA-A*	Major histocompatibility complex, class I, A	HLA-A*02:07:01 HLA-A*30:01:01 HLA-A*33:03 HLA-A*33:03:01 HLA-A*68:01:01:01	carbamazepine valproic acid lamotrigine topiramate	Increased risk of severe cutaneous adverse reactions.	2B	[120,121,122]
*HLA-A*	Major histocompatibility complex, class I, A	HLA-A*31:01:02	carbamazepine *Testing recommended by HCSC and suggested by FDA and PMDA*	Increased risk of severe cutaneous adverse reactions.	1A	[70]
*HLA-B*	Major histocompatibility complex, class I, B	HLA-B*13:02:01 HLA-B*38:01:01	carbamazepine valproic acid lamotrigine topiramate	Increased risk of Stevens–Johnson syndrome, toxic epidermal necrolysis, and maculopapular exanthema.	3	[123,124]
*HLA-B*	Major histocompatibility complex, class I, B	HLA-B*15:02:01	carbamazepine *Testing required by FDA and suggested by PMDA*	Increased risk of Stevens–Johnson syndrome and toxic epidermal necrolysis.	1A	[123,125]
*HLA-C*	Major histocompatibility complex, class I, C	HLA-C*07:18 HLA-C*08:01	carbamazepine valproic acid lamotrigine topiramate	Increased risk of Stevens–Johnson syndrome and toxic epidermal necrolysis.	3	[122]
*HLA-DQB1*	Major histocompatibility complex, class II, DQ beta 1	HLA-DQB1*06:09	carbamazepine valproic acid lamotrigine topiramate	Increased risk of severe cutaneous adverse reactions.	none	[122]
*HLA-DRB1*	major histocompatibility complex, class II, DR beta 1	HLA-DRB1*13:01:01	carbamazepine valproic acid lamotrigine topiramate	Increased risk of severe cutaneous adverse reactions.	none	[122]
*HNF4A*	Hepatocyte nuclear factor 4 alpha	rs2071197	lamotrigine	Patients with the AA genotype may have decreased concentrations of lamotrigine compared to patients with the AG and GG genotypes.	3	[115]
*HRH3*	Histamine receptor H3	rs3787429 rs3787430	risperidone paliperidone	Patients with the TT genotype and schizophrenia may have a better response when treated with risperidone as compared to patients with the CC or CT genotype.	3	[126]
*HRH4*	Histamine receptor H4	rs4483927	risperidone paliperidone	Patients with the TT genotype and schizophrenia may have a poorer response when treated with risperidone as compared to patients with the GT or GG genotype.	3	[127]
*HTR1A*	5-Hydroxytryptamine Receptor 1A	rs6295 rs10042486 rs1364043	antidepressants amisulpride olanzapine quetiapine risperidone	Patients with the CC genotype may have a decreased likelihood of response to antidepressants as compared to patients with the GG or CG genotype.	3	[128,129,130]
				Patients with the TT genotype and schizophrenia may have a better response when treated with antipsychotics, including amisulpride, olanzapine, quetiapine, and risperidone, as compared to patients with the CC or CT genotype.	3	
*HTR1B*	5-hydroxytryptamine receptor 1B	rs130058	clomipramine liothyronine lithium nefazodone venlafaxine	Patients with the AA genotype and depression who are treated with clomipramine, liothyronine, lithium, nefazodone, or venlafaxine may have an increased risk for suicidal ideation as compared to patients with the TT genotype.	3	[25,75]
*HTR2A*	5-Hydroxytryptamine Receptor 2A	rs7997012 rs9567733 rs6314	citalopram antipsychotics antidepressants	Patients with the AA genotype who are treated with citalopram may be more likely to have improvement in symptoms as compared to patients with the GG genotype.	2B	[25,75]
				Patients with the AA genotype and first episode psychosis (FEP) may have a decreased risk for extrapyramidal symptoms when treated with antipsychotics as compared to patients with the AG or GG genotype.	3	
				Patients with the AA genotype who are treated with antidepressants and other treatments may have a reduced response and reduced likelihood of remission as compared to patients with the AG or GG genotype.	3	
*HTR2C*	5-Hydroxytryptamine Receptor 2C	rs1414334 rs3813929	antipsychotics	Male patients with the C genotype and female patients with the CC genotype may have an increased risk of developing metabolic syndrome and weight gain.	2B	[131,132,133,134,135]
				Male patients with the C genotype who are treated with antipsychotics may have an increased risk of weight gain as compared to patients with the T genotype.	2B	
*KCNMA1*	Potassium calcium-activated channel subfamily M alpha 1	rs35793	quetiapine	Allele G is associated with response to quetiapine.	none	[136]
*LEP*	Leptin	rs7799039 rs4731426	risperidone paliperidone olanzapine	Patients with the GG genotype may have an increased likelihood of weight gain when taking antipsychotics.	3	[137,138]
*LEPR*	Leptin receptor	rs1137101	valproic acid antipsychotics	Patients with the GG genotype and epilepsy may have lower weight gain when treated with valproic acid as compared to patients with the AA or AG genotype.	3	[139]
				Female patients with the GG genotype may have an increased likelihood of weight gain when treated with antipsychotics as compared to patients with the AA genotype.	3	
*MC4R*	Melanocortin 4 receptor	rs489693 rs17782313	antipsychotics	Patients with the CC genotype and disorders requiring antipsychotic treatment may have an increased risk of weight gain when treated with antipsychotics as compared to patients with the TT genotype.	2B	[140,141]
				Patients with the AA genotype may have an increased likelihood of weight gain and hypertriglyceridemia when taking antipsychotics as compared to patients with the AC and CC genotypes.	2B	
*MYO1H*	Myosin IH	rs7959663	lithium	Patients with the CC genotype and bipolar affective disorder may have a decreased response to lithium as compared to patients with the CG or GG genotypes.	3	[88]
*NR1D1*	Nuclear receptor subfamily 1 group D member 1	rs2314339 rs2071427	lithium	Patients with the CC or CT genotype and bipolar disorder may be more likely to respond to lithium as compared to patients with the TT genotype.	3	[142,143]
				Patients with the CC genotype and bipolar disorder may be less likely to respond to lithium as compared to patients with the TT genotype.	3	
*NR1I2*	Nuclear receptor subfamily 1 group I member 2	rs7643645 rs2276707	risperidone paliperidone	Patients with the AA genotype may have increased levels of the active metabolite of risperidone, 9-hydroxy-risperidone, as compared to those with the GG genotype.	3	[144,145,146]
				Patients with the CC genotype and psychiatric disorders may have decreased clearance of risperidone compared to patients with the CT or TT genotypes.	3	
*NTRK2*	Neurotrophic tyrosine kinase receptor type 2	rs1387923 rs2769605 rs10465180	lithium valproic acid clozapine	Patients with the AA genotype and bipolar disorder may have increased response to lithium as compared to patients with the AG or GG genotype.	4	[144]
				Patients with the CC genotype and schizophrenia who are treated with clozapine may have a decreased response to clozapine as compared to patients with the CT or TT genotype.	3	
*OR52E2*	Olfactory receptor family 52 subfamily E member 2	rs16909440	lithium	Patients with the CC genotype and bipolar disorder may have a poorer response to treatment with lithium as compared to patients with the CT or TT genotype.	3	[94]
*OTC*	Ornithine carbamoyltransferase	complete gene sequencing	valproic acid *Testing suggested by FDA and PMDA*	Valproic acid is contraindicated in patients with known urea cycle disorders (UCD) due to a risk for severe hyperammonemia. UCDs result from mutations in one of several genes, such as ornithine transcarbamylase (OTC).	none	[102]
*PDE4D*	Phosphodiesterase 4D	rs2164660 rs17382202	quetiapine	Patients with the AA genotype may have an increased response to quetiapine as compared to patients with the AG or GG genotypes.	3	[90]
				Patients with the CC genotype may have a decreased response to quetiapine as compared to patients with the CT or TT genotypes.	3	
*POLG*	DNA polymerase gamma, catalytic subunit	complete gene sequencing	divalprovex valproic acid *Testing required by FDA and HCSC*	Patients with the AA or AT may have an increased risk of hepatotoxicity as compared to patients with the CC genotype.	3	[102]
*PPA2*	Pyrophosphatase (inorganic) 2	rs2636719	risperidone paliperidone	Patients with the CC genotype may have an increased response to risperidone as compared to patients with the AA or AC genotypes.	3	[90]
*RGS4*	Regulator of G protein signaling 4	rs2661319 rs951439	risperidone paliperidone	Patients with the CT genotype treated with risperidone may have more improvement in symptoms as compared to the CC genotype or may have less improvement in symptoms as compared to the TT genotype.	3	[147,148]
*RIMS1*	Regulating synaptic membrane exocytosis 1	rs502046	quetiapine	Genotypes CC + CT are associated with decreased likelihood of discontinuation when treated with quetiapine.	none	[149]
*SCN1A*	Sodium voltage-gated channel alpha subunit 1	rs2298771 rs3812718	carbamazepine phenytoin valproic acid lamotrigine topiramate oxcarbazepine	Patients with the CC genotype who are treated with mono or combination antiepileptic therapy may have an improved response.	3	[150,151,152]
				Patients with the CC genotype who are treated with phenytoin may require a lower dose.	2B	
*SCN2A*	Sodium voltage-gated channel alpha subunit 2	rs17183814 rs2304016	carbamazepine valproic acid lamotrigine	Patients with the GG genotype may be more likely to respond.	3	[153,154]
*SLC18A2*	solute carrier family 18 member 2	rs363224	antipsychotics	Genotypes AC + CC is associated with increased risk of tardive dyskinesia when treated with antipsychotics.	none	[155]
*SLC1A1*	Solute carrier family 1 member 1	rs3780412	clozapine olanzapine risperidone paliperidone	Allele C is associated with increased risk of obsessive-compulsive symptoms when treated with clozapine, olanzapine, and risperidone.	none	[156]
*SLC22A8*	Solute carrier family 22 member 8	rs2276299	risperidone paliperidone	Allele A is not associated with risk of hyperprolactinemia when treated with risperidone in children.	none	[157]
*SLC6A4*	Solute carrier family 6 member 4	SLC6A4 HTTLPR long form (L allele) SLC6A4 HTTLPR short form (S allele)	antidepressants	HTTLPR short form (S allele)/HTTLPR long form (L allele) + HTTLPR short form (S allele)/HTTLPR short form (S allele) is associated with non-response when treated with antidepressants in people with mood disorders as compared to SLC6A4 HTTLPR long form (L allele)/HTTLPR long form (L allele).	2B, 3	[75,93,100]
*TAAR6*	Trace amine associated receptor 6	rs4305746	aripiprazole	Patients with the AA genotype may have faster improvement in brief psychiatric rating scale (BPRS) scores when treated with aripiprazole as compared to patients with the GG genotype.	3	[158]
*TNFRSF11A*	TNF receptor superfamily member 11a	rs2980976	risperidone paliperidone	Patients with schizophrenia and the AA genotype may have a decreased response to risperidone as compared to patients with the AG or GG genotypes.	3	[90]
*TPH1*	Tryptophan hydroxylase 1	rs1799913	lithium	Genotype TT is associated with decreased response to lithium in people with bipolar disorder.	none	[159]
*TPH2*	Tryptophan hydroxylase 2	rs1487278 rs2171363 rs17110747	quetiapine	Patients with the CC or CT genotype may respond better to antidepressant treatments as compared to patients with the TT genotype.	3	[160]
*TYMS*	Thymidylate synthetase	rs3786362	risperidone paliperidone	Allele A is associated with increased risk of hyperprolactinemia when treated with risperidone.	none	[114]
*ZNF804A*	zinc finger protein 804A	rs62200793	lithium	Patients with the CC genotype and bipolar affective disorder may have a decreased response to lithium as compared to patients with the CT or TT genotypes.	3	[88]

**Table 2:** SNPs relevant for pharmacokinetics of drugs used for treatment of bipolar spectrum disorders.

Gene	Protein	SNPs	Drugs	Results	Level of Evidence (1A Strongest)	Ref.
*ABCB1*	ATP binding cassette subfamily B member 1	rs2032582 rs1045642 rs1128503	carbamazepine phenobarbital phenytoin valproic acid amisulpiride aripiprazole olanzapine risperidone paliperidone	Patients with the CC genotype may have decreased risk for non-response as compared to patients with the TT genotype.	3	[164,165,166,167,168]
				Patients with the AA genotype may have decreased concentrations of oxcarbazepine and worse response as compared to patients with the AG and GG genotypes.	3	
				Patients with the AA genotype who responded to treatment with antipsychotics may require a decreased dose of antipsychotics as compared to patients with the CC genotype.	3	
*ABCG2*	ATP binding cassette subfamily G member	rs2231142 rs3114020	risperidone paliperidone lamotrigine	Patients with the CC genotype may have increased concentrations of lamotrigine compared to patients with the TT genotype.	3	[169,170]
*APEH*	Acylaminoacyl-peptide hydrolase	rs3816877	valproic acid divalproex	Genotype CC is associated with increased concentrations of valproic acid in people with Epilepsy as compared to genotype CT.	none	[171]
*CYP1A1*	Cytochrome P450 family 1 subfamily A member 1	rs2606345	valproic acid, divalproex	Female patients with the AA genotype may have a poorer response when treated with antiepileptic drugs as compared to patients with the AC or CC genotype.	3	[172,173]
*CYP1A2*	cytochrome P450 family 1 subfamily A member 2	rs762551	antipsychotics chlorpromazinefluphenazine thioridazine trifluoperazine	Patients with the AA genotype may have decreased QT interval when treated with antipsychotics, chlorpromazine, fluphenazine, thioridazine, and trifluoperazine as compared to patients with genotype CC or AC.	3	[67,174]
*CYP2C19* *	cytochrome P450 family 2 subfamily C member 19	CYP2C19 * 1 CYP2C19 * 17CYP2C19 * 2 CYP2C19 * 3 CYP2C19 * 4	citalopram escitalopram sertraline clomipramine	Patients with the CYP2C19 * 1/* 1 genotype who are treated with citalopram or escitalopram may have an increased drug clearance/metabolism as compared to patients with CYP2C19 * 2, * 3, or * 4 allele and a decreased drug clearance/metabolism as compared to patients with CYP2C19 * 1/* 17 or * 17/* 17 genotype.	1A	[67,175,176,177]
				Patients with the * 1/* 1 diplotype who are treated with sertraline may have lower dose-corrected drug plasma concentrations and increased clearance as compared to patients with one or two CYP2C19 no alleles (* 1/* 2 or * 2/* 2, * 2/* 3).	1A	
				Patients with the CYP2C19 * 1/* 1 genotype may have 1) increased metabolism of clomipramine as compared to patients with CYP2C19 * 2 and * 3 alleles, 2) increased plasma levels of clomipramine as compared to patients with the CYP2C19 * 17/* 17 genotype.	2A	
*CYP2C9*	cytochrome P450 family 2 subfamily C member 9	CYP2C9 * 1 CYP2C9 * 2 CYP2C9 * 3	valproic acid divalproex olanzapine	Patients with the * 1/* 1 genotype and bipolar disorder and other psychotic disorders may have increased dose of valproic acid compared to patients with the * 1/* 2 and * 1/* 3 genotypes.	3	[67,178,179]
				Individuals with the * 1/* 1 genotype were less likely to experience hypotension when receiving olanzapine as compared to individuals with the * 1/* 3, * 2/* 3 or * 3/* 6 genotype.	3	
*CYP2D6* *	cytochrome P450 family 2 subfamily D member 6	CYP2D6 * 1 CYP2D6 * 10 CYP2D6 * 1xNCYP2D6 * 2 CYP2D6 * 2xNCYP2D6 * 3 CYP2D6 * 4 CYP2D6 * 5 CYP2D6 * 6 rs3892097	paroxetine fluvoxamine risperidone clomipramine quetiapine valproic acid divalproex aripiprazole *Testing suggested by FDA, EMA, DPWG and HCSC for aripiprazole, risperidone*	Patients with the CYP2D6 * 1/* 1 genotype who are treated with paroxetine may have (1) a decreased clearance of paroxetine as compared to patients with more than two functional CYP2D6 alleles (* 1xN, * 2xN) and (2) an increased clearance of paroxetine as compared to patients with two non-functional CYP2D6 alleles (* 3, * 4, * 5, * 6) or * 10/* 10 genotype.	1A	[67,180,181,182,183]
				Patients with the CYP2D6 * 1/* 1 genotype who are treated with fluvoxamine may have 1) decreased steady-state plasma concentration-to-dose (C/D) ratio as compared to patients with the * 1/* 5, * 1/* 10, * 5/* 10, * 10/* 10 genotype, 2) decreased plasma concentrations, 3) decreased risk of developing gastrointestinal side effects as compared to patients with the * 5/* 10, * 10/* 10 genotype, and 4) decreased AUC, Cmax and half-life time of fluvoxamine as compared to patients with two non-functional CYP2D6 alleles (poor metabolizer phenotypes).	1A	
				Patients with the * 1 allele may have increased metabolism/clearance of risperidone as compared to patients with two reduced function alleles (* 10), one reduced function and one non-functional (* 4, * 5, or * 14) allele, or two non-functional alleles.	2A	
				Patients with the CC genotype (CYP2D6 * 1/* 1) treated with tricyclic antidepressants (1) may have a decreased likelihood of switching treatment indicating a reduced risk of side effects (2) may require an increased dose of drug as compared to patients with the TT genotype (CYP2D6 * 4/* 4).	1A	
				Patients with the CYP2D6 * 1/* 1 genotype treated with clomipramine may have (1) a decreased, but not absent, risk for side effects as compared to patients with the CYP2D6 * 4 allele, (2) increased plasma concentration of clomipramine and desmethyl clomipramine as compared to patients with a duplication of a functional CYP2D6 gene, (3) decreased plasma concentration of clomipramine and desmethyl clomipramine as compared to patients with two non-functional CYP2D6 alleles.	1A	
*CYP3A4*	cytochrome P450 family 3 subfamily A member 4	rs35599367 rs2242480	risperidone carbamazepine	Patients with the AG genotype may have reduced clearance of risperidone compared to patients with the GG genotype.	3	[67,184,185]
				Patients with the CC genotype (CYP3A4 * 1/* 1) may have increased concentrations of carbamazepine as compared to patients with the CT (* 1/* 1G) or TT (* 1G/* 1G) genotype.	3	
*CYP3A43*	Cytochrome P450 family 3 subfamily A member 43	rs680055	aripiprazole clozapine haloperidol olanzapine quetiapine risperidone	Genotype CG is associated with increased response to antipsychotics, aripiprazole, clozapine, haloperidol, olanzapine, quetiapine, or risperidone in people with schizoaffective disorder or schizophrenia as compared to genotype CC.	none	[186]
*CYP3A5*	cytochrome P450 family 3 subfamily A member 5	rs776746 rs10264272	olanzapine carbamazepine	Individuals with the * 1A/* 1A genotype may have increased area under the curve (AUC) of olanzapine as compared to Individuals with the * 3A/* 3A genotype.	3	[67,187]
				Patients with the CC genotype (CYP3A5 * 3/* 3) may have decreased clearance and increased concentrations of carbamazepine, and require lower doses of the drug, as compared to patients with the CT (* 1/* 3) or TT (* 1/* 1) genotype.	3	
*EPHX2*	epoxide hydrolase 2	rs59724122	lithium	Patients with the CC genotype and bipolar affective disorder may have a decreased response to lithium as compared to patients with the CT or TT genotypes.	3	[88]
*UGT2B7*	UDP glucuronosyltransferase family 2 member B7	rs7438284 rs7668258 rs12233719 rs28365063	valproic acid divalproex lamotrigine oxicarbazepine	Patients with the CC genotype and epilepsy who are treated with valproic acid may have decreased concentrations of valproic acid as compared to patients with the TT genotypes.	3	[188,189,190,191,192,193]
				Patients with the AA genotype and epilepsy may have decreased clearance of lamotrigine compared to patients with the GG genotype.	3	

* see CPIC^®^ Guideline for selective serotonin reuptake inhibitors and CYP2D6 and CYP2C19.
